# One-step Green Fabrication of Antimicrobial Surfaces via In Situ Growth of Copper Oxide Nanoparticles

**DOI:** 10.1021/acsomega.2c02540

**Published:** 2022-07-18

**Authors:** Furkan Sahin, Nusret Celik, Ahmet Ceylan, Mahmut Ruzi, M. Serdar Onses

**Affiliations:** † Nanotechnology Application and Research Center, ERNAM—Erciyes University, Kayseri38039, Turkey; ‡ Department of Materials Science and Engineering, Erciyes University, Kayseri38039, Turkey; § Faculty of Pharmacy, Erciyes University, Kayseri38039, Turkey; ∥ UNAM-Institute of Materials Science and Nanotechnology, Bilkent University, Ankara06800, Turkey

## Abstract

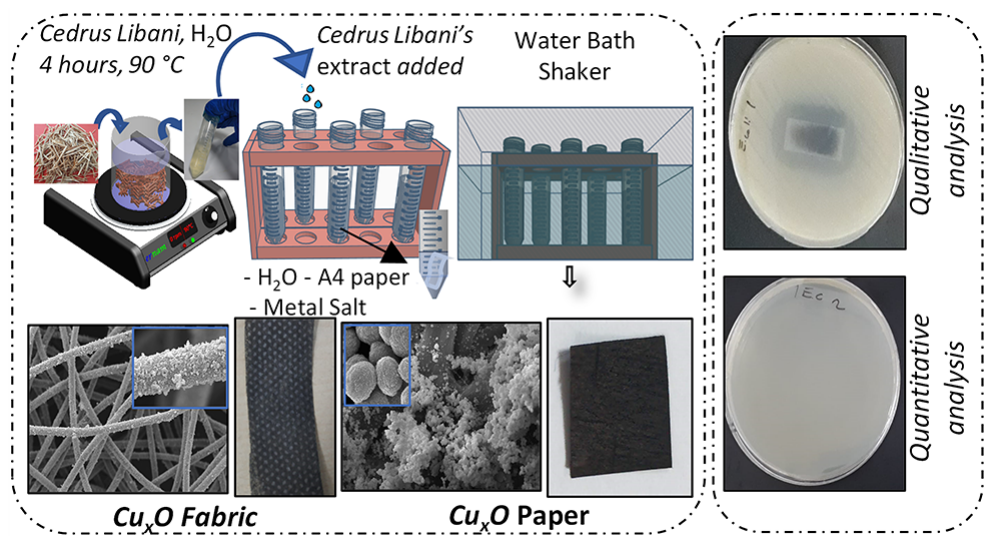

Microorganisms such as pathogenic bacteria, fungi, and viruses pose a serious threat to human health and society. Surfaces are one of the major pathways for the transmission of infectious diseases. Therefore, imparting antipathogenic properties to these surfaces is significant. Here, we present a rapid, one-step approach for practical fabrication of antimicrobial and antifungal surfaces using an eco-friendly and low-cost reducing agent, the extract of *Cedrus libani*. Copper oxide nanoparticles were grown in situ on the surface of print paper and fabric in the presence of the copper salt and extract, without the use of any additional chemicals. The morphology and composition of the grown nanoparticles were characterized using field emission scanning electron microscopy, energy-dispersive X-ray spectroscopy, X-ray photoelectron spectroscopy, and X-ray diffraction techniques. The analysis revealed that the grown particles consist of mainly spherical CuO nanoparticles with an average size of ∼14 nm and its clusters with an average size of ∼700 nm. The in situ growth process enables strong bonding of the nanoparticles to the surface, resulting in enhanced durability against wear and tear. Moreover, the fabricated surface shows excellent growth inhibition ability and bactericidal activity against both gram-negative and gram-positive bacteria, *Escherichia coli* and *Staphylococcus aureus*, as well as antifungal activity against *Candida albicans*, a common pathogenic fungus. The ability to grow copper oxide nanoparticles on different surfaces paves the way for a range of applications in wound dressings, masks, and protective medical equipment.

## 1 Introduction

Microorganisms like bacteria, fungi, and viruses are part of the ecosystem that humans partake, and from time to time, some of the species can cause serious threat to human life. The COVID-19 pandemic is a recent eminent example. Besides the viruses, some bacteria and fungi species such as *Escherichia coli* and *Staphylococcus aureus* have inflicted a severe toll on humanity for centuries. For example, the Global Action Fund for Fungal Infections estimates that more than 300 million people worldwide are infected with fungal infections every year, while more than 1.5 million die from it.^1,2^ Some studies revealed that there is a ∼20% mortality in diseases caused by *E. coli* and *S. aureus*.^3,4^ One of the main hotbeds and routes for the survival and transmission of these pathogenic microorganisms are highly touched surfaces such as portable equipment, bank notes, and door knobs.^5−8^ Regular disinfection of these surfaces and washing hands are conventional methods to mitigate infection. However, the excessive use of chemicals and water is a serious concern from a sustainability perspective. An effective and environment-friendly approach is incorporating antibacterial and antifungal coatings to the surfaces. In this regard, eco-friendly, flexible, and low-cost antimicrobial surfaces are needed.

Due to prominent toxicity to a wide range of pathogenic bacteria and fungus, availability, and low cost, some metals and their oxides, such as Ag, Cu, and Zn, have been used to treat infections since ancient times.^9−11^ Nanoparticles of these materials exhibit enhanced antibacterial activity due to their large surface area, and thus various physical and chemical methods have been developed to fabricate antibacterial metal/oxide nanoparticles.^12,13^ However, these conventional methods lead to environmental pollution, which is another worldwide problem and therefore necessitates the development of antibacterial surfaces employing eco-friendly materials and processes. In this aspect, green synthesis based on plants and their extracts to reduce metal salts comes to the rescue due to their wide availability and renewability and the environmental friendliness of waste products.^14,15^ Phytochemicals and biological molecules such as natural alkaloids, phenolics, flavonoids, glycosides, terpenoids, enzymes, and amino acids are abundant in plant extract and can act as reducing, capping, and stabilizing agents.^16−18^ These characteristics allow particles of various shapes and sizes to be fabricated conveniently and with high efficiency.^19,20^

Metallic copper and its oxides are cheap and thus an excellent candidate for fabricating antibacterial surfaces employing green methods, as demonstrated by recent studies.^21−27^ Most of these studies follow a similar route: obtaining plant extracts (mostly leaves) and using this extract to reduce metal salts to prepare a colloidal nanoparticle solution, followed by applying these colloidal nanoparticles onto a surface to obtain the final, functional surface. A more direct and robust approach would be to grow the nanoparticles in situ on the desired surface, which can provide a heterogeneous nucleation site to the reduced metal atoms and may reduce the nucleation barrier, thereby enhancing the efficiency. However, there are only a few limited studies on the direct growth of functional nanoparticles on surfaces, mostly on porous materials such as cellulose, cotton, and polyester. Nonetheless, these previous studies demand thermal treatment at high temperatures^22^ and usage of corrosive and toxic chemicals^23^ and generally require multiple steps.^25^ Furthermore, very few studies investigated the robustness of those antipathogenic surfaces, and even those focused only on washing tests related to antibacterial fabrics.^21,28^ However, in real-life applications, mechanical abrasion due to touch and scratch are inevitable, and thus the resistance of green fabricated antimicrobial surfaces to abrasion should also be demonstrated.

Herein, we present a practical approach to preparing oxide nanoparticles of copper with very high antimicrobial and antifungal activities in one step using an aqueous extract of *Cedrus libani*, a widely cultivated tree native to the Eastern Mediterranean and is stated to have antibacterial/antifungal ability in folk medicine. The chemical composition and structure of the in situ-grown nanoparticles are characterized, followed by investigating their antibacterial and antifungal activities. The results indicate that the main advantages of the presented approach are the usage of eco-friendly and low-cost materials, which are suited for mass production and mechanical durability.

## 2 Results and Discussion

**Figure 1:**
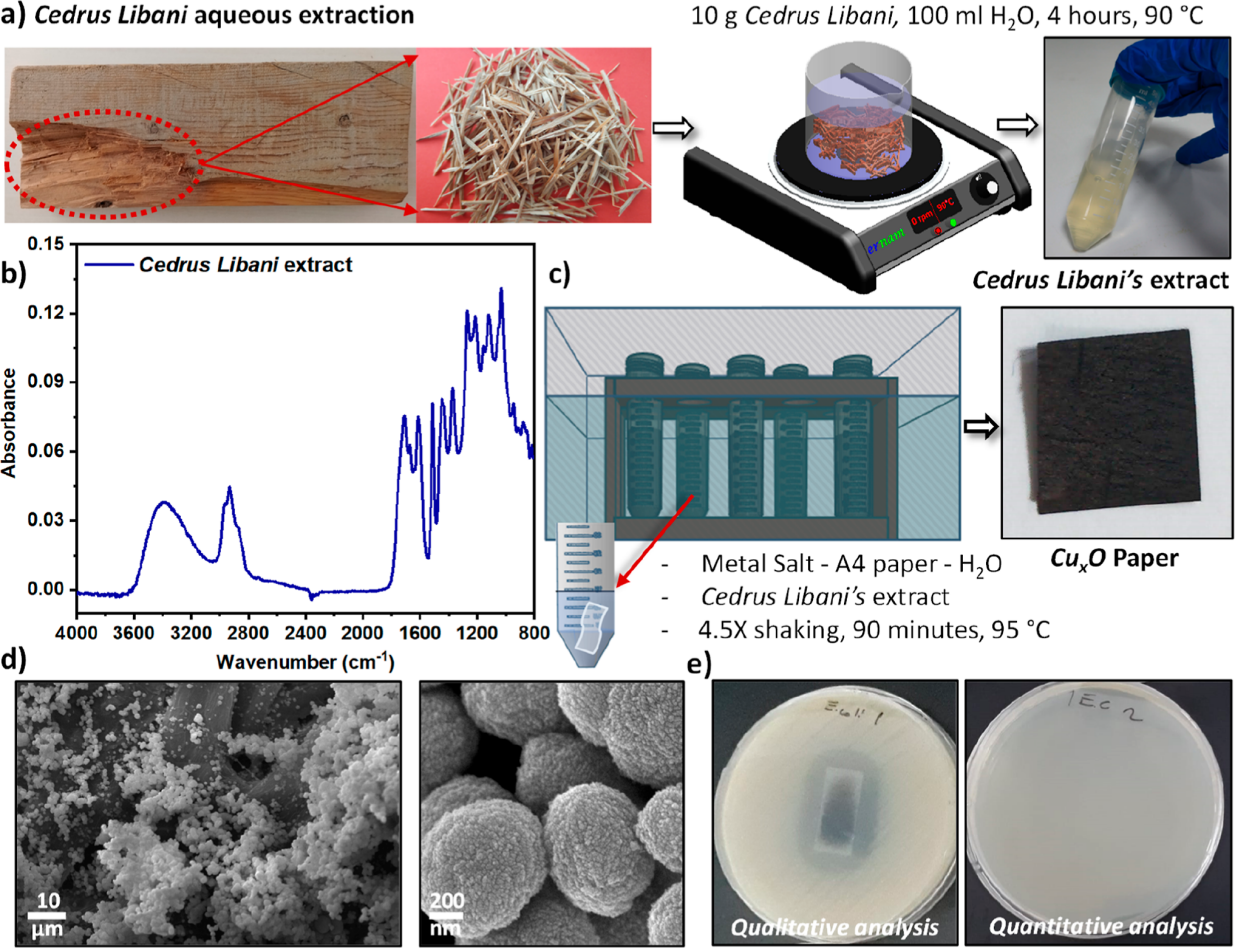
Fabrication and characterization of the antibacterial surface. (a) Photographs of the *C. libani* wood and pieces, an illustration of the extraction process, and a photograph of the extract in a test tube. (b) FTIR–ATR spectrum of the dried extract. (c) Illustration of the reaction setup for reducing the metal salt using the extract under heating and shaking. Also shown is the photograph of the paper after the growth of nanoparticles. (d) FESEM images of the Cu_*x*_O paper surface at different magnifications. (e) Example of the antimicrobial activity of the resultant surfaces against *E. coli*, showing the zone of inhibition against *E. coli* (left) and bactericidal activity (right). No viable bacterial colonies are visible on the agar plate (right).

This study presents facile fabrication of antibacterial surfaces using an eco-friendly approach. Specifically, a natural extract from *C. libani* was used for reducing metal salts instead of toxic and expensive chemicals.^29^ Furthermore, we also resort to reducing the metal salts on a surface, print paper to be specific. The advantages of growing the nanoparticles on a surface are one-step production, low cost, homogeneity of the surface, and resistance to physical abrasion. Shown in Figure 1a are pictures of *C. libani* and illustrations of the extraction process. Since water is used, only molecules with enough solubility in water are expected in the aqueous extract (pale yellow). The Fourier transform infrared (FTIR) spectrum (Figure 1b) of the dried extract can help shed light on the chemical nature of the components. The strong and broad peak at around 3400 cm^–1^ is due to the O–H stretch vibration and indicates multiple OH groups. The peak at around 2940 cm^–1^ is due to the C–H stretch of alkane groups. The strong and sharp peaks at 1700, 1600, and 1500 cm^–1^ indicate the C=O stretch of carboxylic groups, the C–C stretch of benzene rings, and the bending vibrations of amino groups, respectively. Besides, there are multiple and complex peaks in the fingerprint region due to the C–O stretch and the C–H bending vibrations. Overall, the FTIR spectra suggest the existence of polyphenols and tannins in the aqueous extract, in agreement with previous studies on plant extracts.^30,31^ These compounds are known to be able to reduce the metal salts.

The prepared aqueous extract was then mixed with a metal salt in a test tube, and then a piece of paper was immersed into the mixed solution, followed by heating the test tube with a water bath at 95 °C for 90 min. At the end of the reaction, the color of the print paper turns black as a result of the growth of nanoparticles on the surface (Figure 1c). Finally, the antibacterial properties of the surfaces were evaluated by following the protocols of modified ISO 22196 (test for antimicrobial activity) and disk diffusion methods against Gram-positive *S. aureus* and Gram-negative *E. coli* bacteria (Figure 1e).

**Figure 2:**
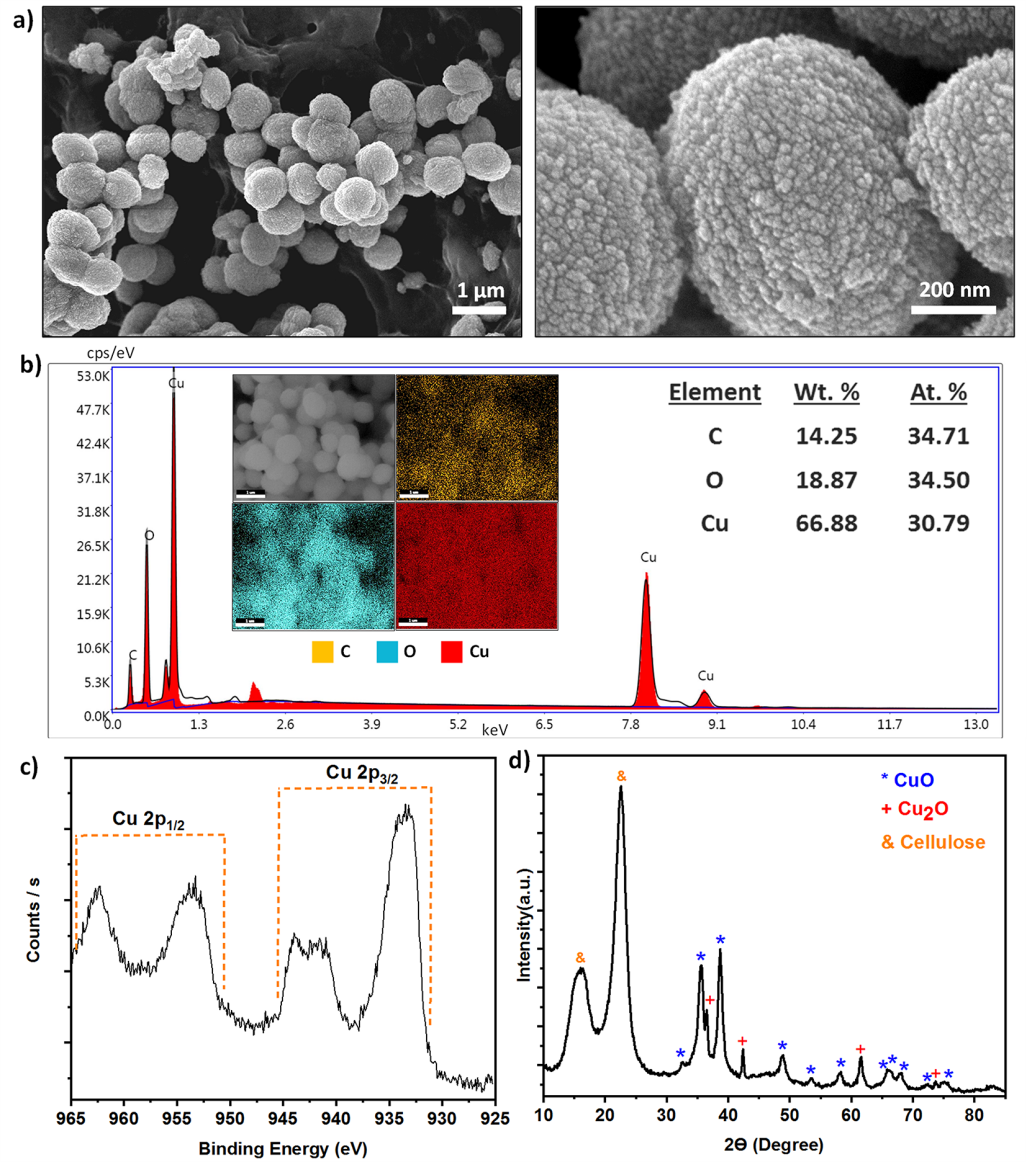
Structural and chemical analyses of Cu_*x*_O paper. (a) FESEM images of Cu_*x*_O paper at the magnification of 10,000 (left) and 100,000 (right). (b) EDX elemental analysis and mapping (inset) of the Cu_*x*_O paper surface. (c) High-resolution XPS spectra of the Cu_*x*_O paper surface around the Cu 2p region. (d) XRD diffraction pattern of the Cu_*x*_O paper surface.

The structure and morphology of the prepared surfaces were further analyzed using field emission scanning electron microscopy (FESEM), energy-dispersive X-ray spectroscopy (EDX) mapping, and X-ray diffraction (XRD). Figure 2a shows the FESEM images of the surface, showing that the Cu_*x*_O paper sample surface consists of particles of different scales. The large ones have an average diameter of 0.7 ± 0.1 μm (Supporting Information Figure S1a). On top of the large particles, nanoparticles with an average diameter of 14 ± 4 nm (*n* = 100) (Supporting Information Figure S1b) are formed. These large particles are likely to be clusters of the primary nanoparticles.

The chemical composition of metallic structures on the surface was investigated by EDX elemental analysis and mapping (Figure 2b). The results show that the ratio of copper atoms to oxygen atoms is close to 1, which indicates the formation of CuO nanoparticles. The EDX mapping suggests a homogeneous surface, which can be explained by the microroughness of the paper providing abundant nucleation sites.^32−34^ To better understand the chemical structure of the copper oxide nanoparticles, X-ray photoelectron spectroscopy (XPS) was performed. The XPS survey spectrum consists of characteristics peaks of Cu 2p, O 1s, and C 1s (Supporting Information Figure S2). Furthermore, high-resolution XPS spectra of Cu around the 2p region identifies the chemical state of Cu. A typical XPS spectrum around the Cu 2p region is shown in Figure 2c, where main peaks appear at 933.4 eV (Cu 2p_3/2_) and 953.9 eV (Cu 2p_1/2_), along with their two shake-up satellites peaks.^35^ These satellites show strong configuration interactions and are specific for identifying copper(II).^36^ The spectral features of Cu 2p_1/2_ and Cu 2p_3/2_ peaks (Supporting Information Figure S2a) agree with previous studies.^35−38^ However, the peaks seemed to be influenced by copper(I) such as in Cu_2_O (Supporting Information Figure S2b,c). In the XPS spectra, the binding energies of Cu(I) and Cu(II) are so close and not differentiable under current experimental conditions, and therefore the surfaces prepared in this study likely contain both species.

To examine the crystal structure of the grown nanoparticles, an XRD analysis was performed. As shown in Figure 2d, the XRD pattern exhibits characteristic peaks at 2θ = 32.6, 35.7, 38.7, 48.8, 53.4, 58.2, 61.6, 65.8, 66.2, 68.1, 72.1, and 75.3° corresponding to the (110), (002, 1̅1̅1), (111, 200), (2̅0̅2), (020), (220), (1̅1̅3), (022), (3̅1̅1), (220), (311), and (004, 2̅2̅2) planes of the CuO (JCPDS #no. 05-0661).^39^ Additionally, the pattern indicates a crystallized structure due to the peaks observed at 2θ = 36.5, 42.4, 61.6, and 73.7°, which can be assigned to the (111), (200), and (311) crystallographic faces of Cu_2_O (reference JCPDS card no. 75-1531).^40,41^ As expected, cellulose-specific peaks also appear at 2θ = 15.7 and 22.6°.^42^ No other characteristic peaks were observed in the XRD pattern, confirming the high purity of the particles that were synthesized on the surface. Furthermore, the profile of the XRD peak of the CuO nanoparticles due to diffraction from the (111) plane is useful for calculating the primary particle size using the Debye–Scherrer equation (eq 2). The calculated particle size is 9.8 nm, which is close to the particle size of 14 nm extracted from the FESEM images.

**Figure 3:**
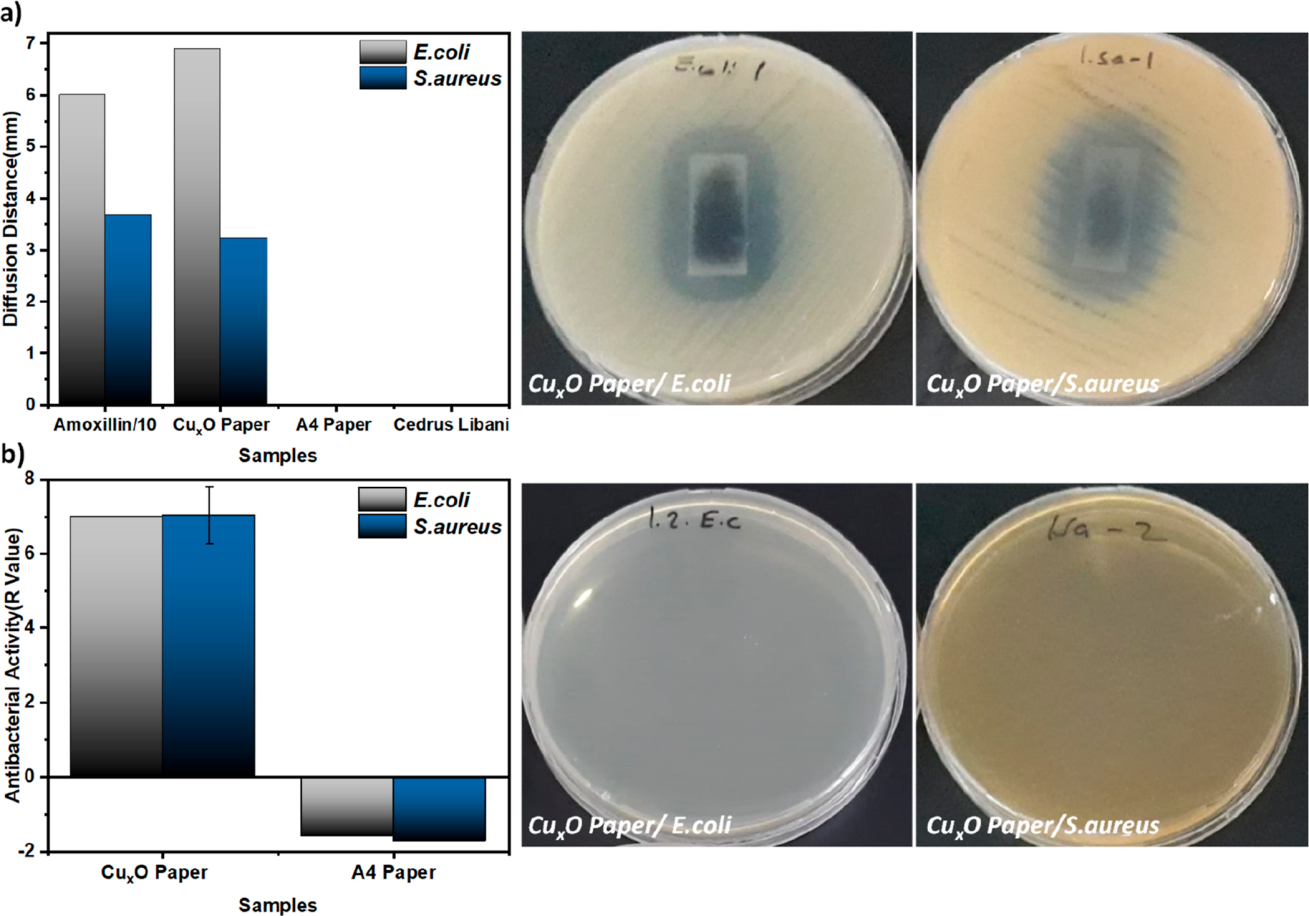
Evaluation of the antibacterial activity of the prepared Cu_*x*_O paper. (a) Bacterial growth inhibition ability of various samples. Left: diffusion distances and right: photographs of agar plate showing the diffusion disk results. (b) Bactericidal activity of the prepared surface. Left: antibacterial activity (*R*-value); right: pictures of agar plate showing killing test for *E. coli* (left) and *S. aureus* (right).

To evaluate the antibacterial activity of the copper oxide nanoparticles grown on the paper surface, two of the most common bacteria, *E. coli* and *S. aureus,* were chosen as test targets. Specifically, the inhibition ability of the fabricated surfaces was assessed qualitatively by calculating the inhibition diameters with the parallel streak method, and the bactericidal activity was evaluated with the modified AATCC 100 method by calculating the kill log rate, as shown in eq 1. As controls, we also evaluated the antimicrobial activity of the print paper and *C. libani* aqueous extract. As shown in Figure 3a, the print paper and *C. libani* aqueous extract did not show any antibacterial activity, while positive control amoxicillin/10 antibiotic showed an inhibition diameter of 6 mm against *E. coli* and approximately 0.4 mm against *S. aureus* (see Supporting Information Figure S3 for more details). On the other hand, the fabricated Cu_*x*_O paper samples showed impressive antibacterial activity with inhibition diameter zones of 6.9 mm against *E. coli* and 3.2 mm against *S. aureus* (Figure 3a). Interestingly, Cu_*x*_O papers exhibited the same antibacterial activity and inhibition diameter even on a small substrate area (1 × 1.5 cm^2^) (Supporting Information Figure S4 and Table S1). This may be the result of a dense growth of metallic nanostructures on the surface. Overall, the antibacterial effect of Cu_*x*_O paper can be described as good.^43^ These results agree with previous studies where Cu, with the advantages of being oxidized easily, high solubility, and high ion release rate, shows impressive antibacterial activity.^44−46^

To evaluate the antibacterial effect of green-fabricated surfaces quantitatively, a colony of 2.5 × 10^5^*E. coli* and 5.6 × 10^5^*S. aureus* were cultivated on the surfaces, and the bactericidal efficacy of the surfaces was quantified by colony counting after 24 h of incubation. The fabricated Cu_*x*_O samples showed complete killing for *E. coli* and *S. aureus* (Figure 3b). On the other hand, the microorganisms increased approximately 100-fold on untreated paper, proving that pure paper had no antibacterial activity (Table S2 and Figure S5). The prepared Cu_*x*_O paper is hydrophilic (water contact angle is 34°, see Figure S6) which is beneficial for increasing the contact surface area via facilitating the spreading of the bacterial solution, thus increasing bactericidal activity.

**Figure 4:**
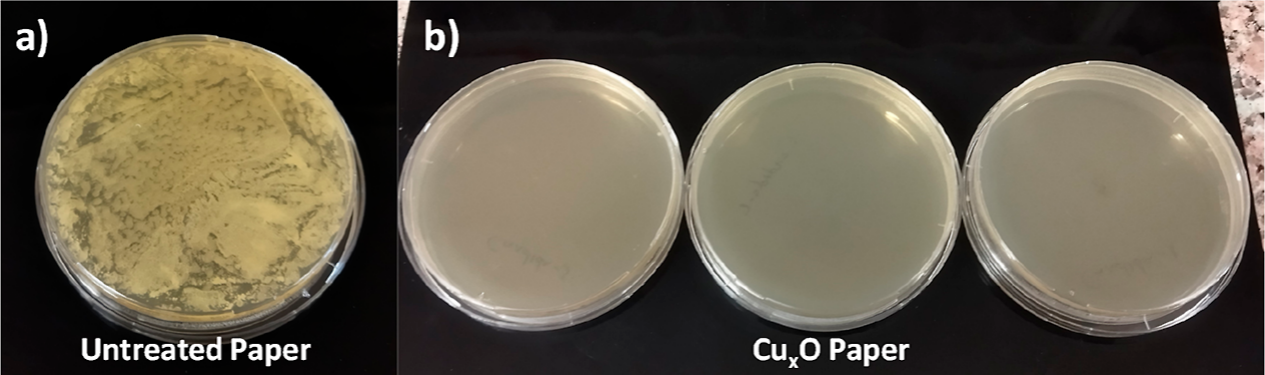
Antifungal activity of Cu_*x*_O paper. (a) Proliferation of *C. albicans* on the untreated paper surface. (b) Agar plate photos showing Cu_*x*_O paper killing all *C. albicans* (*n* = 3).

Besides the two common bacteria, *Candida albicans* is a fungal species that causes serious health concerns as it is the leading cause of nosocomial infections, especially among immunocompromised individuals.^47,48^ Motivated by this challenge, we examined the antifungal properties of the Cu_*x*_O paper. Figure 4 shows the results of the fungicidal test. The Cu_*x*_O paper substrates completely killed the fungi placed on it (antifungal activity *R* = 7), while the fungi grew aggressively on the untreated paper sample (control). One of the possible mechanisms of the high antifungal activity of the surface is fungal apoptosis induced by oxidative stress via increasing reactive oxygen species activation of copper oxide particles.^49^ Copper oxide particles can also diffuse into the cell via ionic channels and purines, causing leakage of the cytoplasm, deforming the nuclear membrane, and damaging DNA processes.^50^ In addition, it is also possible that the nanoscale spherical particles increase the antimicrobial effect due to the increased surface area/volume ratio.

**Figure 5:**
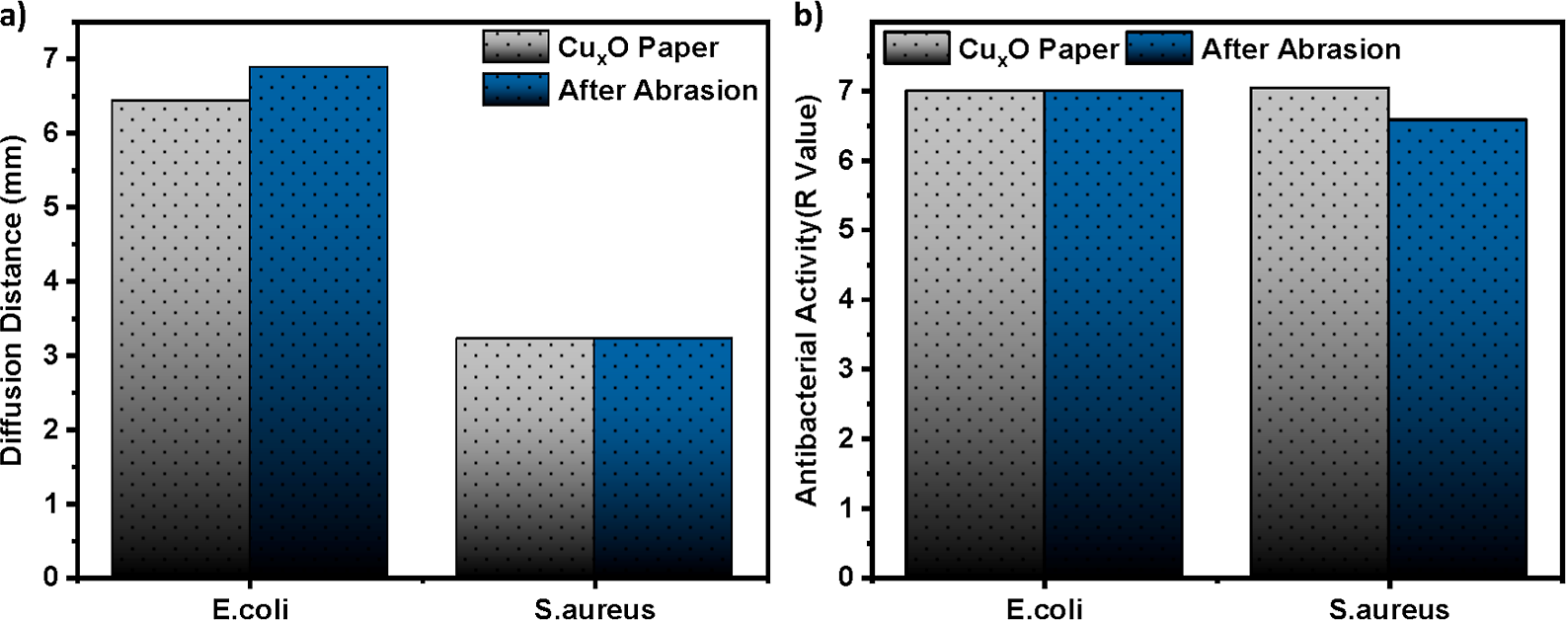
Abrasion resistance of green fabricated antimicrobial Cu_*x*_O paper for practical applications. (a) Change of growth inhibition ability and (b) bactericidal activity of the antibacterial surface before and after abrasion.

The high antibacterial and antifungal activities of the nanoparticles grown on paper surfaces show great promise for applications in the hygiene and healthcare sectors. To evaluate the feasibility of the surface in practical applications, the fabricated Cu_*x*_O paper surface was abraded, and its antibacterial activity after abrasion was examined. As shown in Figure 5, the average diffusion distance for *E. coli* and *S. aureus* after abrasion was 6.44 and 3.22 mm, while the logarithmic reduction antibacterial activity value was 7 and 6.6, respectively (see details in Supporting Information Figures S7 and S8). This durability test clearly shows that the green fabricated surfaces maintain their antibacterial activity even after mechanical abrasion.

**Figure 6:**
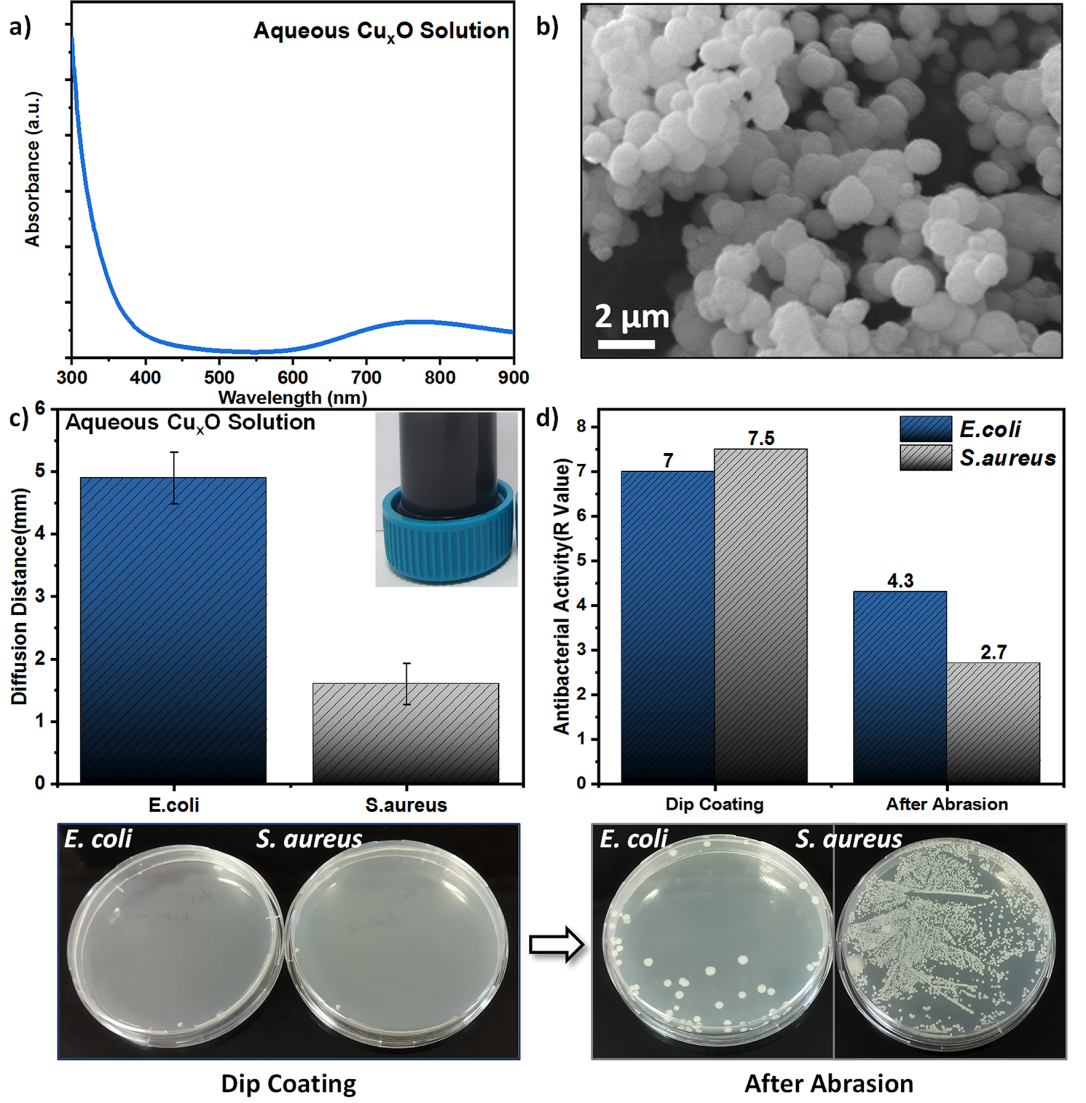
Colloidal synthesis of Cu_*x*_O nanoparticles and evaluation of the antibacterial activity. (a) UV–vis spectra of the Cu_*x*_O nanoparticle solution. (b) SEM images of the print paper after dip coating with Cu_*x*_O nanoparticle solution. (c) Disk diffusion results of green synthesized Cu_*x*_O particles (*n* = 3). (d) Bactericidal activities of the print paper surface coated with colloidal copper oxide nanoparticles and postabrasion surface (*n* = 2). The Petri plate photographs show the bactericidal activity of the Cu_*x*_O surface fabricated via dip coating and after abrasion.

To further investigate the advantage of the in situ growth strategy, we synthesized copper oxide nanoparticles under the same conditions (without paper) and deposited them on the paper surface via dip coating. First, we characterized the synthesized copper oxide nanoparticle solution by measuring the zeta potential (Supporting Information Figure S9) and the UV–vis spectra. As shown in Figure 6a, the UV–vis spectra show absorbance maximum at ∼300 nm which is due to the interband transition in CuO. A weak band is also visible at around 750 nm, which is due to unreacted Cu^2+^ ions.^51^ To prepare a sample, the synthesized copper oxide nanoparticles were dip-coated onto a piece of print paper for characterization. The SEM image of the dip-coated paper surface shows spherical particles (Figure 6b) with an average size of 1093 nm (Supporting Information Figure S9b). Furthermore, the antibacterial activity of the synthesized colloidal nanoparticles was evaluated. The disk diffusion test was performed by placing 10 μL of the synthesized aqueous Cu_*x*_O solution onto blank discs, and the bactericidal activity against both bacterial species was thus qualitatively confirmed (Figure 6c and Supporting Information Figure S10). Dip coating reveals that the deposition of the synthesized Cu_*x*_O nanoparticles on the paper surface is not homogenous (Supporting Information Figure S11). Surprisingly, these dip-coated surfaces showed extremely good bactericidal activity. However, the durability of the dip-coated surfaces is weak where the bactericidal activity is significantly decreased after abrasion (Figure 6d).

**Figure 7:**
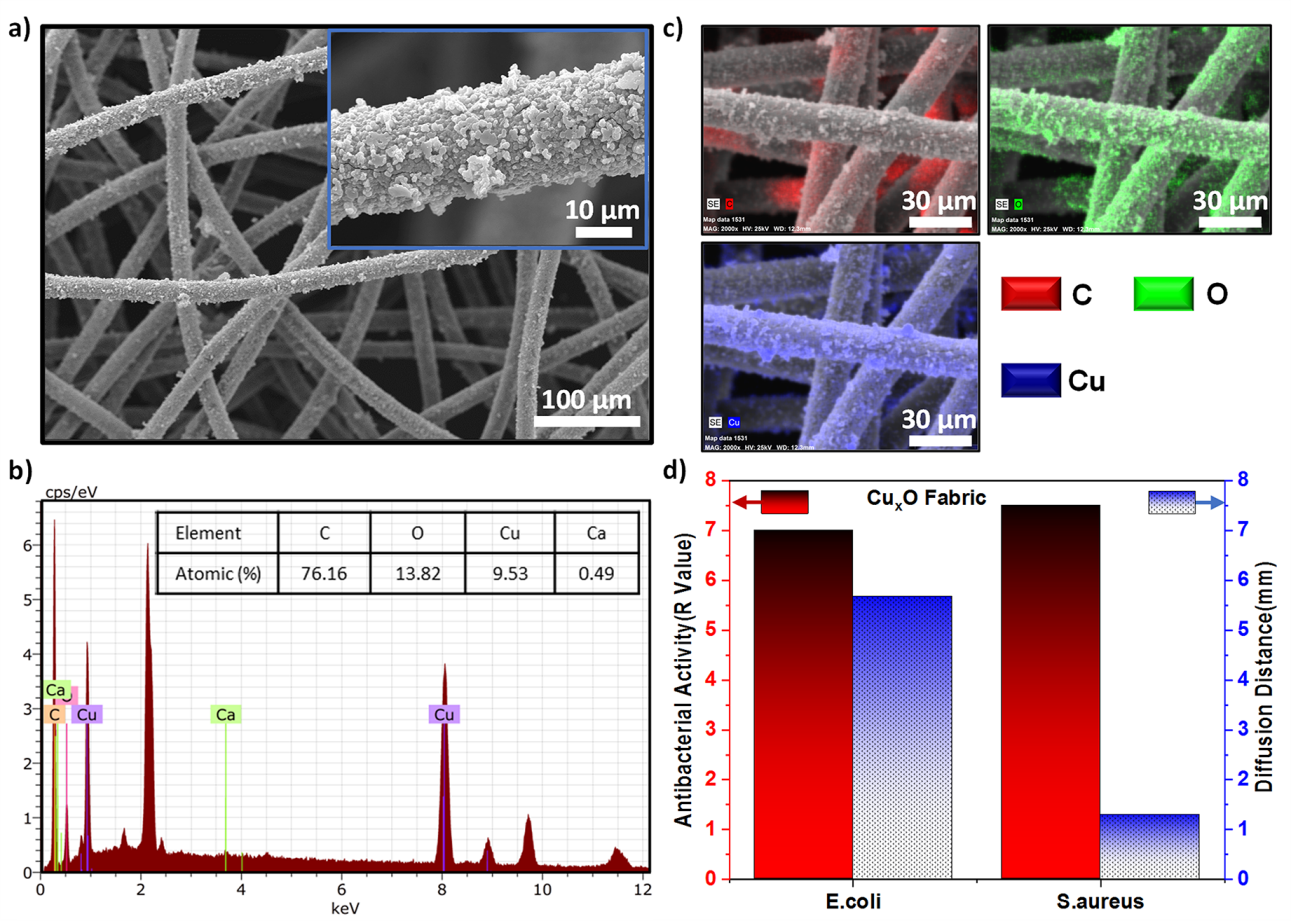
Demonstrating the applicability of the copper oxide particle growth method on different surfaces. (a) SEM images of the Cu_*x*_O fabric surface at 500 magnifications. (b) Elemental mapping of the Cu_*x*_O fabric. (c) EDX elemental analysis of the Cu_*x*_O paper. (d) Qualitative and quantitative antibacterial activities of copper oxide particles grown on the fabric.

Most importantly, we anticipate that postabrasion antibacterial stability is an advantage of the in situ growth strategy. This makes our surfaces promising for practical applications in the hygiene and healthcare sectors. Encouraged by the strong antibacterial activity and mechanical stability, we have grown the Cu_*x*_O sample on a commercial fabric (used for fabricating masks) under the same conditions to show that it can be used for wound dressing, mask, gauze, and so forth (Figure 7 and Supporting Information Figure S12). The SEM image and EDX elemental mapping of the prepared fabrics presented in Figure 7a,b show that the nanoparticles grow densely on the fibers of the fabric. The fact that the surface contains Cu/O in a ratio of about 1:1, as on the Cu_*x*_O paper surface, is an indication that the nanoparticles are copper oxide (Figure 7c). As a result of this, the Cu_*x*_O fabric showed antibacterial activity against both *E. coli* and *S. aureus*, with zones of inhibition of 5.7 and 1.3 mm in diameter, respectively. As shown in Figure 7d, the green fabricated Cu_*x*_O fabric killed all bacteria on it, while on the untreated fabric, the viability of the bacteria increased approximately 100 times (Supporting Information Figures S13 and S14). Overall, the antibacterial characteristics of the prepared Cu_*x*_O fabric are comparable with those of the recently reported Cu/Zn coated cotton mask fiber.^52^ The bactericidal effect on all green fabricated surfaces is probably the result of nano/micro metallic particles that encounter the bacteria, breaking down the bacterial membrane, infiltrating the cell, or damaging the protein/DNA structures.^53,54^

## 3 Conclusions

This study demonstrated the practical fabrication of antimicrobial and antifungal surfaces using a plant extract mediated synthesis of Cu_*x*_O nanoparticles. The aqueous extract was prepared from a widely cultivated plant trunk, and chemical analysis revealed the existence of polyphenols which can easily reduce metal salts. Employing the plant extract and metal sources, copper oxide nanoparticles were successfully grown on print paper and fabrics. These textured surfaces not only provide nucleation sites to accelerate the growth of particles but also enhance the binding of the grown particles to the surface, thus increasing durability against mechanical wear. The most important advantage of the in situ growth strategy is that durable antimicrobial surfaces can be fabricated in a single step with low-cost, abundant materials. The growth of copper oxide particles on the surface of the paper and mask fabric imparted these surfaces’ high lethality against *C. albicans*, *E. coli*, and *S. aureus* pathogens. The fact that the presented platform can be applied to different surfaces brings flexibility to antimicrobial application areas. Furthermore, green fabricated surfaces showed good stability against mechanical abrasion. This, along with the possibility of eco-friendly, sustainable, and simple production, brings it one step closer to using it in real-life applications.

## 4 Materials and Methods

### 4.1 Preparation of Aqueous Extract

Kindling wood of *C. Libani* was collected from the Taurus Mountains near Anamur district, Mersin, Turkey. A chunk of the *C. libani* wood was cut into small pieces, and 10 g of the *C. libani* pieces were placed in a beaker, followed by adding 100 mL of double-distilled water. Afterward, the beaker was heated at 90 °C for 4 h and then cooled to room temperature. Consequently, the solid content was filtered out with the aid of a filter paper (Macherey-Nagel, MN 640 m, diam. 125 mm), resulting in approximately 60 mL of *C. libani* aqueous extract.

### 4.2 Growth of Metallic Nanostructures on Surfaces

Print paper or fabric (Evony) was cut into small pieces (1 × 3 cm) and placed in a test tube, followed by sequentially adding 15 mL of distilled water, 100 mg of metal salt [Cu(CO_2_CH_3_)_2_·H_2_O, Sigma-Aldrich], and 3 mL of aqueous *C. libani* extract. Afterward, the test tube was placed in a water bath (Memmert WNB14) that was kept at 95 °C, and the content was mixed via shaking at 4.5× for 1.5 h. After that, the surfaces on which the metallic nanostructures were grown (paper or fabric) were retrieved and left to dry at room temperature. For brevity, the prepared sample is called Cu_*x*_O paper (copper oxides).

For comparison, Cu_*x*_O colloid nanoparticles were synthesized under the same conditions following the same procedures for dip coating of surfaces; then a piece of paper was fixed to the bottom of a Petri dish and kept in 15 mL of aqueous copper oxide solution for 1.5 h at room temperature.

### 4.3 Antibacterial Assay

To evaluate the antibacterial activity of the samples, Gram-negative *E. coli* (ATCC25922) and Gram-positive *S. aureus* (ATCT25923) were used. For quantitative analysis, the AATCC 100 test protocol was followed with slight modification. Specifically, 0.5 McFarland suspension of bacteria was prepared in peptone water, followed by adding a broth (Mueller Hinton) in the ratio of 1/9 (bacteria suspension/broth, v/v). Then, 100 μL of the prepared broth mixture was withdrawn and spread on the prepared sample surface. The samples were kept in a 100 mL flask in an incubator (Innova 42, New Brunswick Scientific) at 37 °C and 85% humidity for 24 h. After that, the samples were retrieved and washed in 10 mL of phosphate buffer solution (PBS, pH = 7.4, Sigma-Aldrich) by sonicating for 10 min and vortexing for 1 min. After washing, 100 μL of the bacterial suspension was taken from the flask and spread onto an agar plate kept in the Petri dish. For *E. coli*, nutrient agar (Merck) was used, and for *S. aureus*, tryptic soy agar (Merck) was used. The number of colonies was counted after keeping the Petri dishes in an incubator at 37 °C for 24 h. Since the bacterial colonies in the control samples grew very densely, the bacterial suspension of the control samples was serially diluted 10^2^, 10^4^, and 10^6^ times after washing and then grown in Petri dishes to be able to count the colonies. The antibacterial activity was quantified using *R* values, as calculated according to eq 1
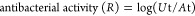
1Here, *U*t is the average bacterial colony number obtained from control samples, while *A*t is the average bacterial colony number obtained from prepared surfaces.

To qualitatively measure the ability of the prepared surfaces to inhibit the growth of bacteria, the AATCC 147 parallel streak method was followed. Specifically, a cotton swab was dipped once in a 0.5 McFarland bacterial suspension, and then parallel streak cultivation was done so that there was no space on the agar. The inhibition zone diameter was measured after 24 h of incubation. To compare the inhibition effect of the samples, amoxicillin/10 antibiotic was used as a positive control.

### 4.4 Antifungal Assay

*C. albicans* was used to evaluate the antifungal activity of the samples. The fungal suspension was prepared in Sabouraud-2% dextrose broth (Merck). Then, 100 μL of the prepared suspension was taken and spread on the Cu_*x*_O paper. For comparison, the antifungal activity of untreated paper was also evaluated and used as the control group. The samples were kept in a cabinet at 37 °C and 85% humidity conditions for 36 h. It was then washed in PBS as in the bactericidal test procedure. After washing, 100 μL of the PBS suspension was taken and spread on Sabouraud 4% dextrose agar (Merck). After 24 h of incubation, fungal colonies on agar were counted, and antifungal activity was calculated according to eq 1.

### 4.5 Robustness of the Antibacterial Surfaces

The robustness of the antimicrobial surfaces was evaluated using an abrasion test. Specifically, as shown in our previous work,^55^ the prepared surfaces were placed under 200 g of weight and moved 100 cm against an aluminum foil. The abrasion test simulates wear and erosion of surfaces which may degrade the antibacterial performance. Therefore, antibacterial activity after abrasion was also evaluated following the same procedures as outlined in the previous section.

### 4.6 Characterization

An FTIR microspectrometer (LUMOS II, Bruker) was used to characterize the *C. libani* extract. Specifically, a few droplets of aqueous extract were placed on a piece of clean aluminum foil and left to dry. Then, FTIR spectra were measured between 680 and 4000 cm^–1^ at a spectral resolution of 4 cm^–1^ and a scan number of 30 using ATR configuration on five different spots, and the average spectrum was presented.

Surface morphology and chemical composition of the prepared surfaces were characterized using SEM (Zeiss EVO LS10), FESEM (Zeiss Gemini 500), and EDS (Bruker) at 25 keV. The size distribution of surface particles was calculated from the SEM images using ImageJ. The wettability of prepared surfaces was evaluated by measuring the static contact angle of a water droplet (10 μL) using a contact angle goniometer (Attension, Theta Lite). XPS was used to analyze the surface composition of the prepared samples using a photoelectron spectrometer (K-alpha, Thermo Scientific) equipped with a monochromatic Al Kα X-ray source (1486.7 eV). Thin-film XRD analyses were performed using a Panalytical Empyrean diffractometer operating at 40 kV and 30 mA using a Cu Kα radiation source. The average size of crystallites was obtained from the XRD data using the Debye–Scherrer eq 2.
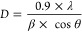
2where θ, λ, and β are the Bragg’s angle of the peaks, the wavelength of X-ray radiation, and the angular width value of peaks at full width at half-maximum, respectively.

## Supporting Information Available

Characterization of Cu_*x*_O nanoparticles and full results of bactericidal and bacterial growth inhibition tests (PDF)
