# Supplementary material for: One-step Green Fabrication of Antimicrobial Surfaces via In Situ Growth of Copper Oxide Nanoparticles
Source: ACS Omega. 2022 Jul 18;7(30):26504–13. doi: 10.1021/acsomega.2c02540 (PMC9352341; doi:10.1021/acsomega.2c02540)
Supplement: Supplementary file 1 — ao2c02540_si_001.pdf [file ao2c02540_si_001.pdf]

One-step green fabrication of antimicrobial surfaces via in situ growth of copper oxide nanoparticles

Furkan Sahin [a], Nusret Celik [a, b], Ahmet Ceylan [c], Mahmut Ruzi [a*], M. Serdar Onses [a ,b, d*]

[a] ERNAM - Erciyes University Nanotechnology Application and Research Center, Kayseri 38039, Turkey

[b] Department of Materials Science and Engineering, Erciyes University, Kayseri 38039, Turkey

[c] Faculty of Pharmacy, Erciyes University, Kayseri 38039, Turkey

[d] UNAM−Institute of Materials Science and Nanotechnology, Bilkent University, Ankara 06800, Turkey

* Corresponding author at: ERNAM – Erciyes University Nanotechnology Application and Research Center, Kayseri 38039, Turkey.

E-mail addresses: mruzi17@gmail.com (MR), onses@erciyes.edu.tr (MSO)

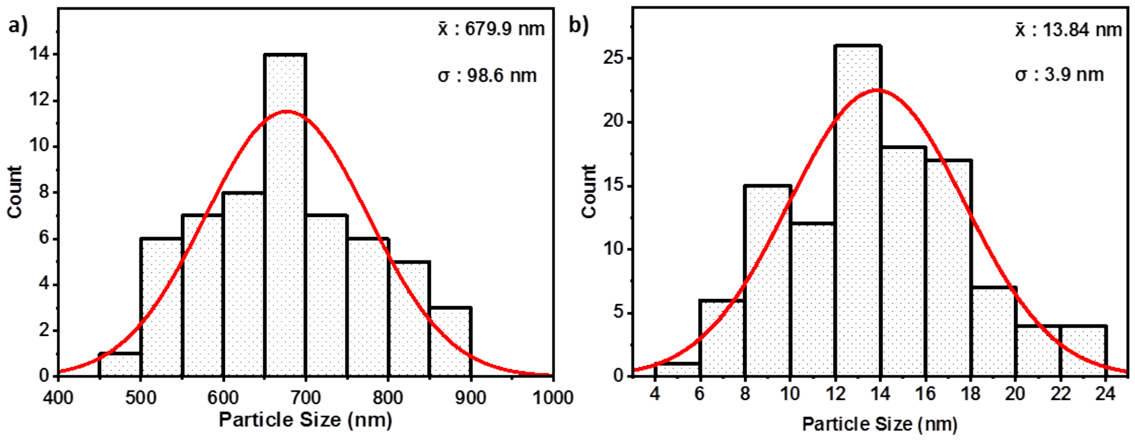

Figure S1. The size distribution of Cu_xO nanoparticles calculated from FESEM images: a) Micro particles b) primary particles that form the micro particles.

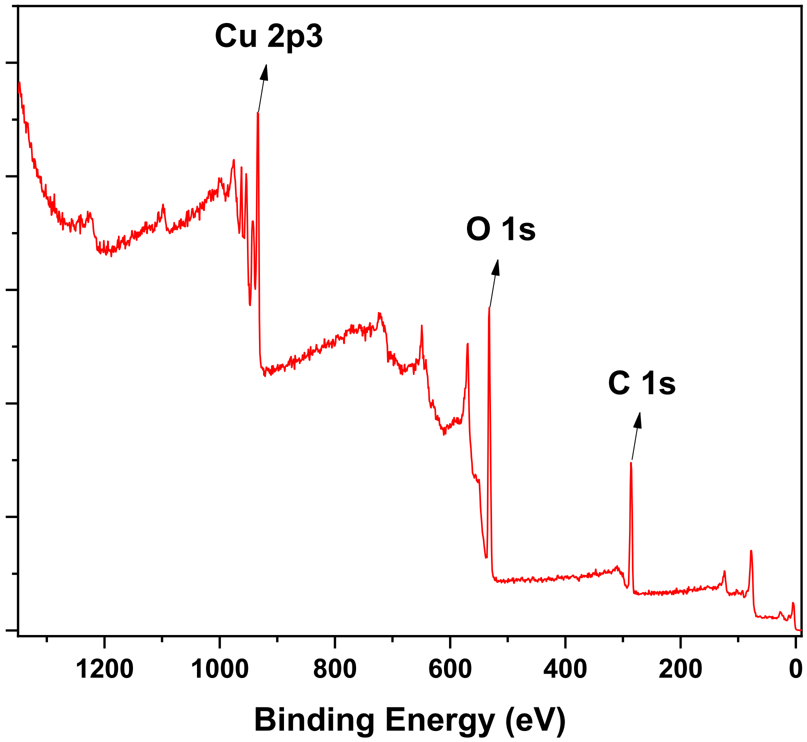

Figure S2. XPS survey spectrum of the in situ grown nanoparticles.

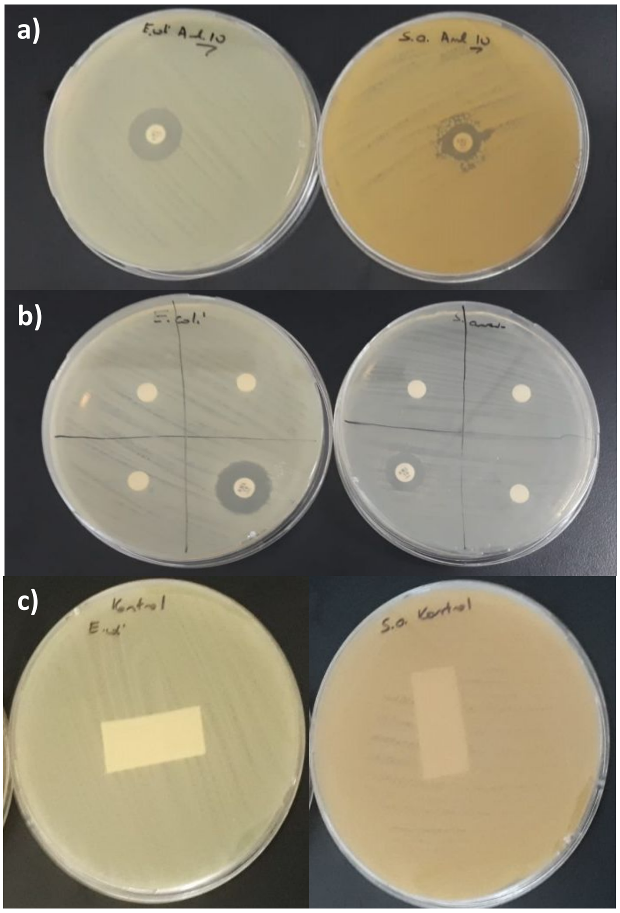

Figure S3. Qualitative evaluation of antibacterial activity of control groups. Shown are disk diffusion results of a) Amoxicillin/10 positive control group, b) *C. libani* aqueous extract, and c) a piece of A4 paper. For all samples, the right petri dish show *S. aureus* experiments while the left petri dishes show *E. coli* experiments.

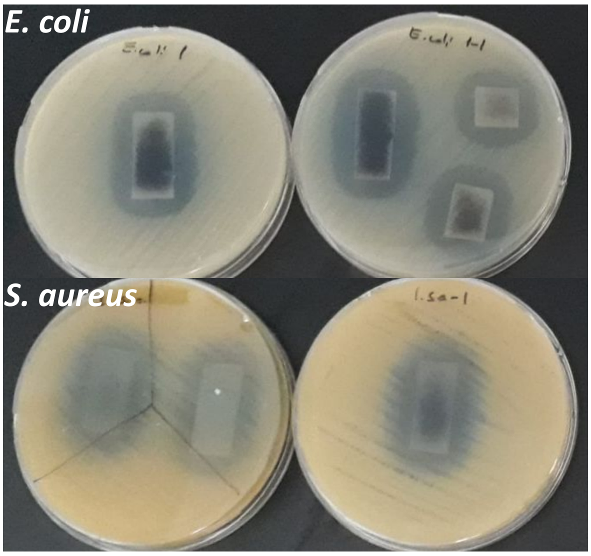

Figure S4. Results of bacterial growth inhibition ability of Cu_xO paper (n=3). It should be noted that Cu_xO paper has the same inhibition diameter even at smaller sizes (top left).

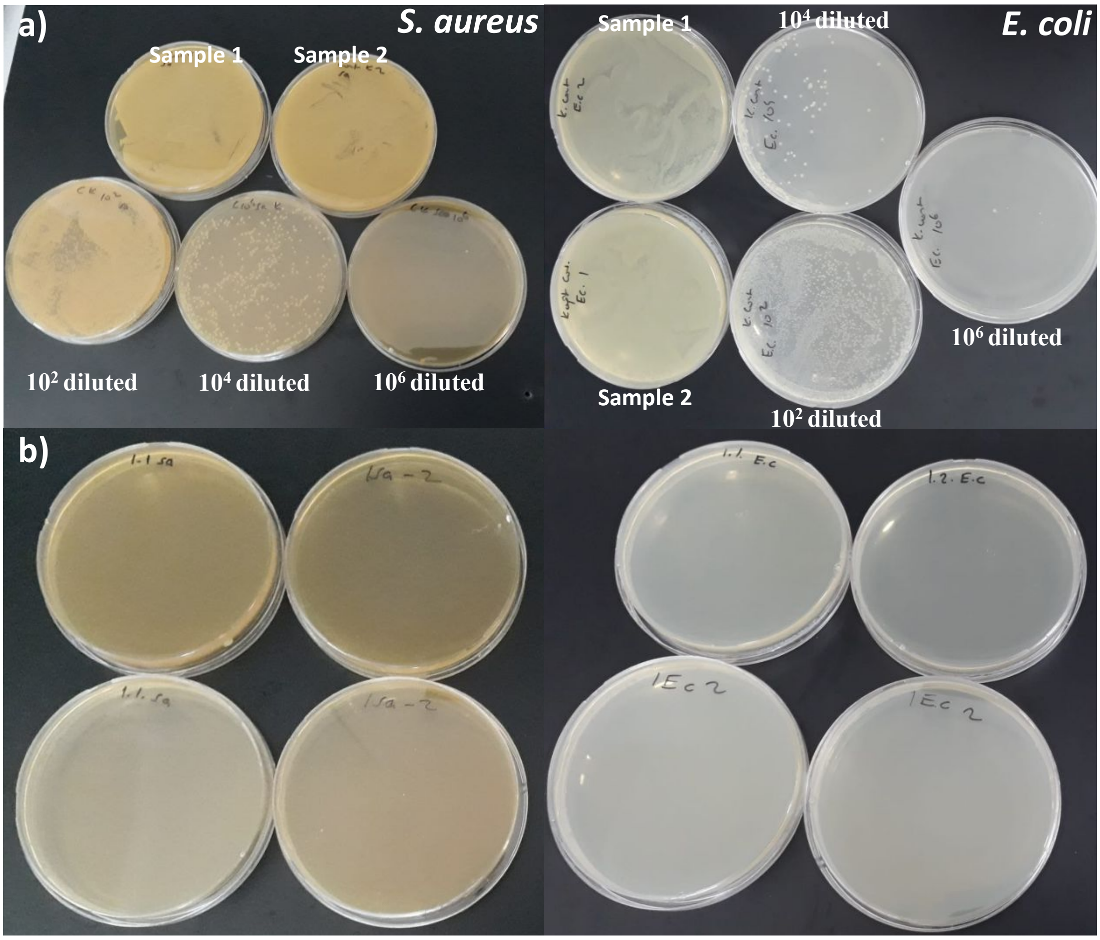

Figure S5. Bactericidal activity of control surface and the fabricated surface. a) bacterial growth results for *S. aureus* and *E. coli*, respectively, on untreated paper (n=2). Experiments were repeated twice with serial dilution of 2,4,6 order of magnitude was applied for colony counting. b) Bacterial growth results for *S. aureus* and *E. coli*, respectively, on Cu_xO paper (n=4).

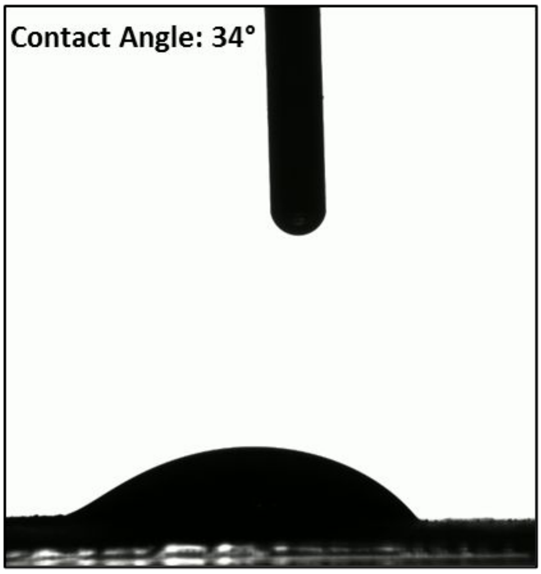

Figure S6. Static contact angle of Cu_xO paper.

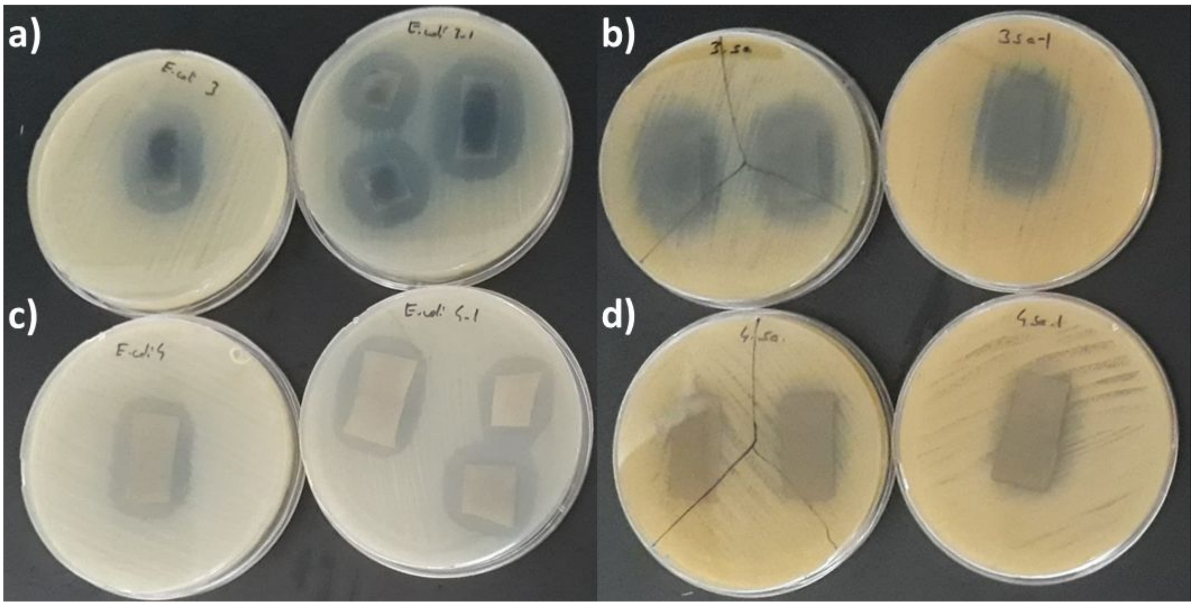

Figure S7. Top row: diffusion results of the abraded Cu_xO paper against *E. coli* (a) and *S. aureus* (b). Bottom row: diffusion results of copper oxide grown fabric against *E. coli* (c) and *S. aureus* (d).

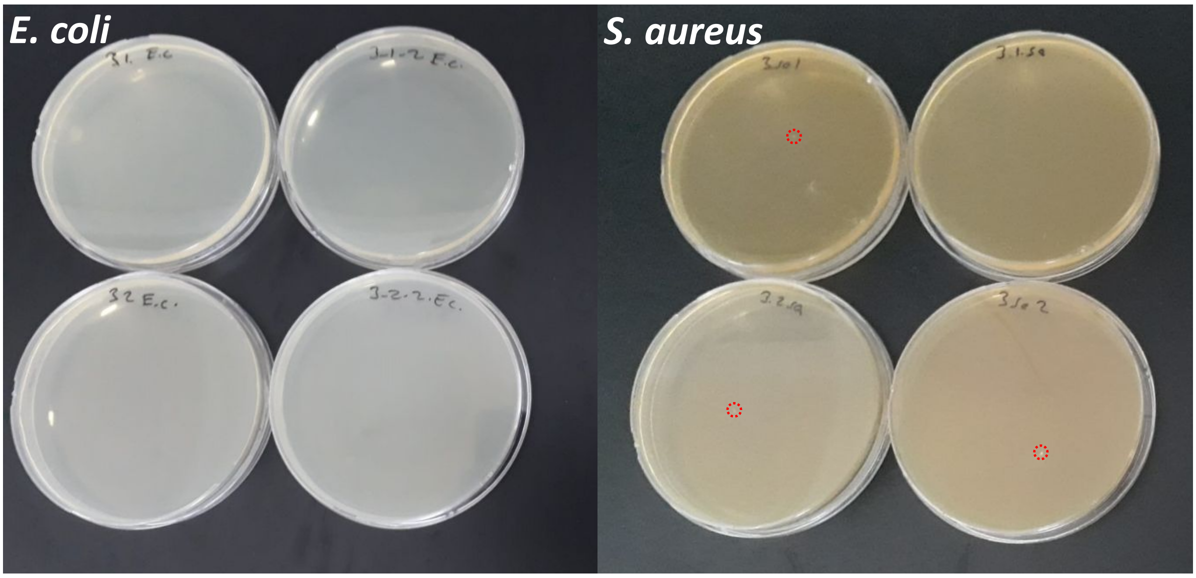

Figure S8. Bactericidal activity of Cu_xO paper after abrasion (n=4) against *E. coli* (left) and *S. aureus* (right).

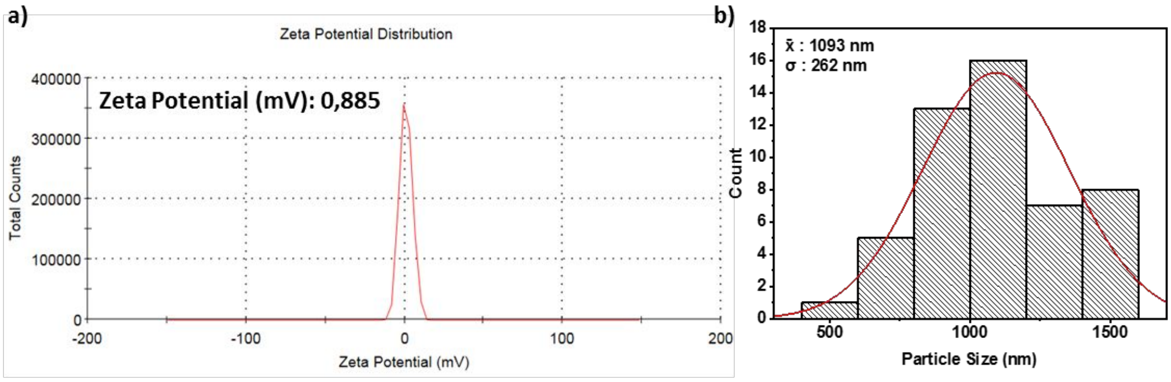

Figure S9. Characterization of green synthesized copper oxide particle solution. a) Zeta potential of aqueous copper oxide particle solution. b) Particle size distribution extracted from the SEM image using ImageJ.

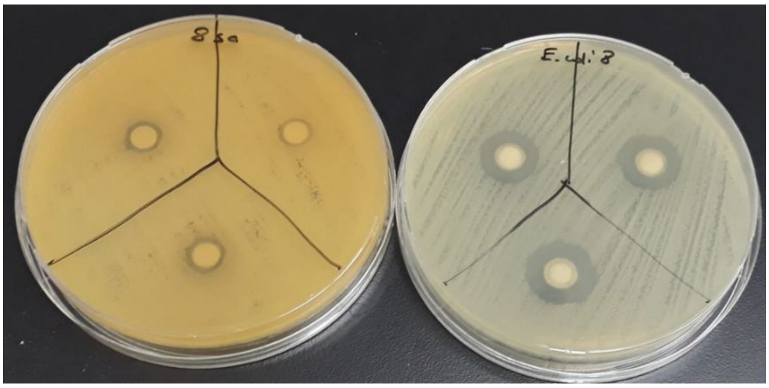

Figure S10. Disk diffusion results of 10 µl aqueous Cu_xO solution against *S.aureus* (left) and *E.coli* (right). The tests were performed three times.

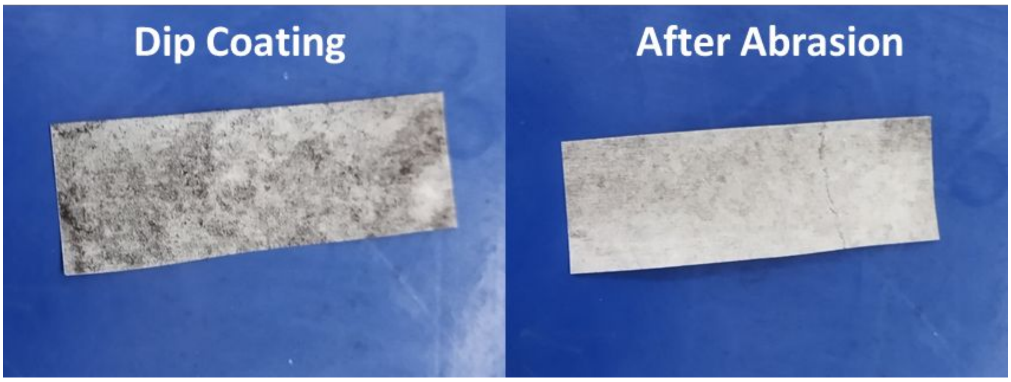

Figure S11. A4 paper surface dip-coated with the synthesized colloidal solutions of nanoparticles and after abrasion.

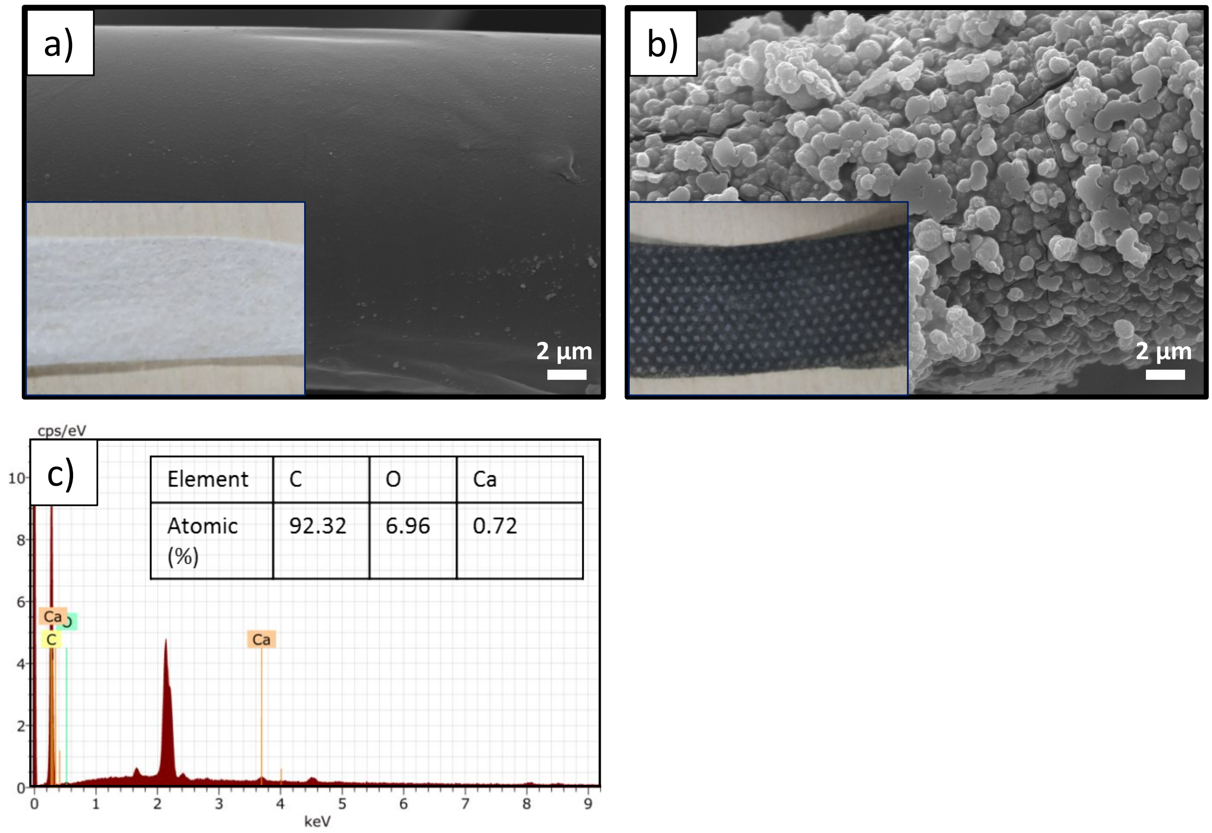

Figure S12. a) SEM images of pristine fabric. b) SEM images of Cu_xO fabric. c) Elemental analysis of pristine fabric. The insets in the bottom right of a-b are the photographs of the corresponding materials.

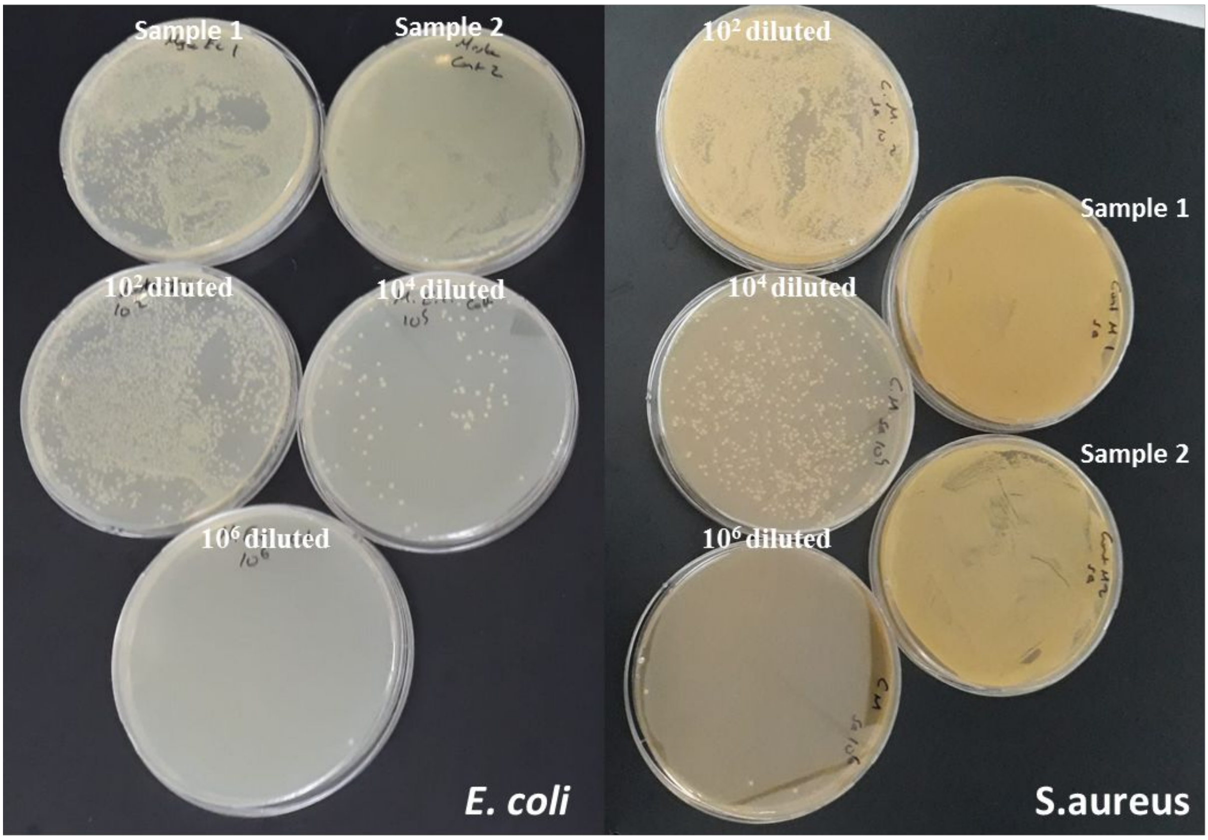

Figure S13. Bacterial growth results for *E. coli* and *S. aureus*, respectively, on untreated fabric (n=2). Experiments were repeated twice with serial dilution of 2,4,6 order of magnitude was applied for colony counting.

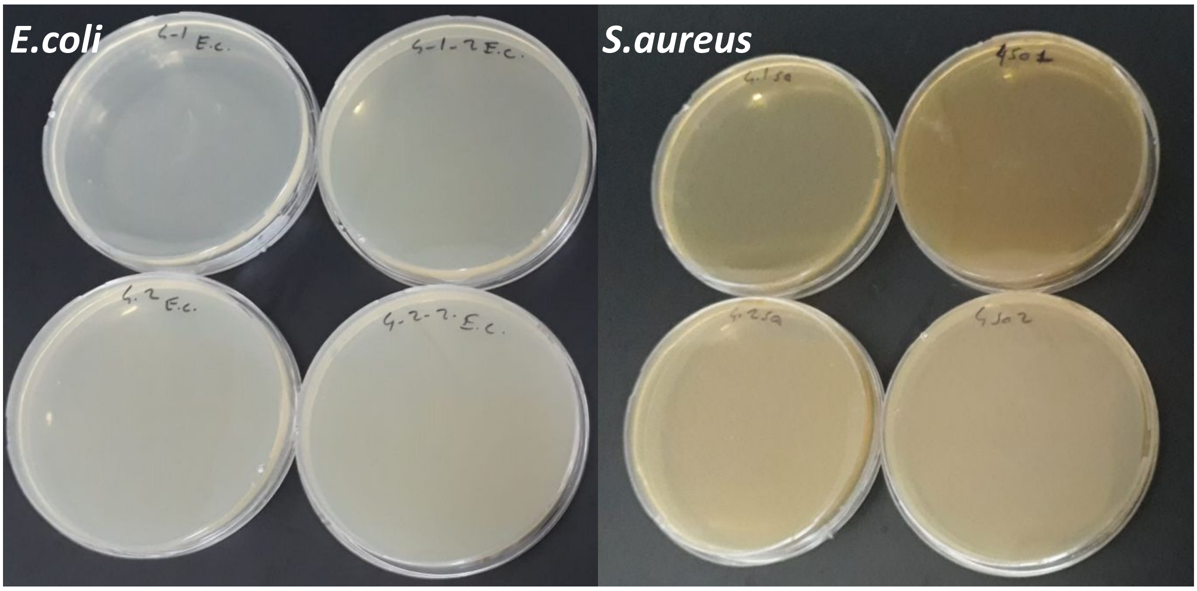

Figure S14. Bactericidal activity of Cu_xO fabric (n=4).

Table S1. The zone of inhibition diameter results of all experiments (Diffusion test results)

Sample name	Diffusion Distance After 24 Hours	
	E. coli	*S. aureus*
**Amoxillin/10**	0.6 cm, 0.6 cm, 0.6 cm	0.5 cm, 0.3 cm, 0.3 cm
**Cedrus Libani**	0	0
**A4 Paper**	0	0
**Cu_xO Paper**	0.8 cm, 0.8 cm, 0.8 cm<br>0.8 cm, 0.6 cm, 0.5 cm<br>0.8 cm, 0.6 cm, 0.5 cm	0.3 cm, 0.3 cm, 0.3 cm<br>0.3 cm, 0.3 cm, 0.2 cm<br>0.2 cm, 0.5 cm, 0.5 cm
**Cu_xO Paper- After Abrasion**	0.6 cm, 0.7 cm, 0.8 cm<br>0.8 cm, 0.7 cm, 0.6 cm<br>0.6 cm, 0.5 cm, 0.5 cm	0.5 cm, 0.3 cm, 0.2 cm<br>0.5 cm, 0.3 cm, 0.5 cm<br>0.3 cm, 0.2 cm, 0.1 cm
**Aqueous Cu_xO Solution**	0.5 cm, 0.5 cm, 0.5 cm<br>0.5 cm, 0.4 cm, 0.5 cm<br>0.5 cm, 0.5 cm, 0.5 cm	0.15 cm, 0.1 cm, 0.2 cm<br>0.2 cm, 0.2 cm, 0.2 cm<br>0.1 cm, 0.1 cm, 0.15 cm

Table S2. Bactericidal test results of all surfaces

Sample name	Coloni Units After 24 hours (cfu/ml, n=4)		Number of initial colonies (cfu/ml)		Antibacterial Activity R=Control(24h)-Sample(24h) (log10)	
	E. coli	*S. aureus*	*E. coli*	*S. aureus*	*E. coli*	*S. aureus*
**Paper**	1×10^7	3×10^7	2.5×10^5	5.6×10^5	-	-
**Cu_xO Paper**	0<br>0<br>0<br>0	0<br>0<br>0<br>60	2.5×10^5	5.6×10^5	7	7.035
**Cu_xO Paper – After Abrasion**	0<br>0<br>0<br>0	10<br>0<br>20<br>20	2.5×10^5	5.6×10^5	7	6.58
**Cu_xO Fabric**	0<br>0<br>0<br>0	0<br>0<br>0<br>0	2.5×10^5	5.6×10^5	7	7.5
**Fabric**	1×10^7	3×10^7	2.5×10^5	5.6×10^5	-	-
